# Genetic context of *bla*_CTX–M–55_ and *qnrS1* genes in a foodborne *Salmonella enterica* serotype Saintpaul isolate from China

**DOI:** 10.3389/fmicb.2022.899062

**Published:** 2022-08-09

**Authors:** Lili Li, Rikke Heidemann Olsen, Jian Xiao, Hecheng Meng, Shifu Peng, Lei Shi

**Affiliations:** ^1^Institute of Food Safety and Nutrition, Jinan University, Guangzhou, China; ^2^School of Food Science and Engineering, South China University of Technology, Guangzhou, China; ^3^Department of Veterinary and Animal Sciences, Faculty of Health and Medical Sciences, University of Copenhagen, Copenhagen, Denmark; ^4^Guangzhou Food Inspection Institute, Guangzhou, China; ^5^Department of Environment and Health, Jiangsu Center for Disease Control and Prevention, Nanjing, China

**Keywords:** *S*. Saintpaul, plasmid, *bla*_*CTX–M*–55_, *qnrS1*, fish

## Abstract

*Salmonella enterica* resistant to fluoroquinolones (FQs) and extended-spectrum cephalosporins (ESCs) has been deemed a high-priority pathogen by the WHO. *Salmonella enterica* serovar Saintpaul (*S*. Saintpaul) co-resistant to ESCs and FQs and harboring corresponding resistance genes (*bla*_CTX–M–55_ and *qnrS1*) have been previously reported. However, they have not been reported in China. Moreover, the genetic context and transferability of ESCs and FQs resistance genes in *S*. Saintpaul remain obscure. This study is the first study to characterize a multidrug-resistant (MDR) *S*. Saintpaul isolate (16Sal016) harboring plasmid-mediated *bla*_CTX–M–55_ and *qnrS1* genes recovered from weever fish in China. The whole genome short- and long-read sequencing results identified the presence of 15 acquired antibiotic resistance genes encoding resistance to nine classes of antibiotics, as well as abundant mobile genetic elements residing on a 259,529 bp IncHI2 plasmid. The *bla*_CTX–M–55_ and *qnrS1* genes were located in a 12,865 bp region, IS*26*-*orf*-*orf*-IS*Kpn19-qnrS1*-IS*3*-Tn*3*-*orf*-*bla*_CTX–M–55_-IS*Ec9*-*orf*-IS*26*. Similar structures have been identified in various bacterial species, indicating a high transferability of *bla*_CTX–M–55_ and *qnrS1* genes within this gene cluster. The plasmid was found to be transferable to *Escherichia coli* (*E. coli*) J53 by conjugation and resulted in the acquisition of multiple resistances by the transconjugants. Genome sequence comparisons by core genome multilocus sequence typing (cgMLST) based on global 2,947 *S*. Saintpaul isolates indicated that strain 16Sal016 was epidemiologically linked with an isolate from the United Kingdom (UK). Our findings suggest that plasmids and IS*26*-mediated mobile genetic elements are carriers of *bla*_CTX–M–55_ and *qnrS1* genes in *S*. Saintpaul, and highlight their potential transmission, which needs continuous investigations.

## Introduction

*Salmonella* is a leading cause of foodborne illness worldwide (Chen et al., 2020). Over the past two decades, multidrug-resistant (MDR) *Salmonella* strains have emerged in clinical settings and food products. These strains, especially those resistant to extended-spectrum cephalosporins (ESCs) and fluoroquinolones (FQs), are concerning because they encode resistance to critically important antibiotic classes commonly used to treat severe salmonellosis (Lu et al., 2019; Zhang et al., 2019).

*Salmonella enterica* serovar Saintpaul (*S*. Saintpaul) is an important gut pathogen, which has been reported to be associated with several outbreaks in many countries (Klontz et al., 2010; Hayford et al., 2015). It was among the top 20 most common serovars observed in the United States (CDC, 2021), and was reported as the second most common cause of outbreaks in Australia, 2001–2016 (Ford et al., 2018), as well as was noted to be among the top five most common serovars in Singapore in 2015 and 2016 (Aung et al., 2020). In China, *S*. Saintpaul has been reported in pets, seafood, poultry, and samples of human origin (Gong et al., 2014; Qi et al., 2019; Zhan et al., 2019; Wang et al., 2020; Wei et al., 2020). MDR resistance has been reported in *S*. Saintpaul isolates; however, co-resistance to ESCs and FQs is uncommon and has not been reported in China (Nadimpalli et al., 2019; Octavia et al., 2021). In China, various *Salmonella* serovars have been reported to acquire steadily increasing co-resistances to clinically important antibiotics (cefotaxime and ciprofloxacin) (Zhan et al., 2019). Consequently, it is important to pay concern to strains of *S*. Saintpaul acquiring those resistances as they may greatly limit current treatment options.

In this study, we characterized an ESCs- and FQs-resistant *S*. Saintpaul isolate recovered from a weever fish in Guangzhou, China, and analyzed the genetic context of corresponding resistance genes, their transferability, as well as the origin, in order to gain insight into their public health impact.

## Materials and methods

### Strains isolation and identification

During our routine surveillance of foodborne pathogens on various food products, *Salmonella* isolate, named GSJ/2016-*Sal*.-016 (hereafter 16Sal016), was recovered from a weever fish (*Lateolabrax japonicus*) in the Guangdong Province, China. The fish sample was collected from a retail market in Guangzhou, Southern China. The isolate was first identified by biochemical confirmation using API 20E test identification test strips (bioMérieux, France), and then 16S ribosomal RNA (rRNA) gene sequencing using the universal primers 27F (5′-AGAGTTTGATCCTGGCTCAG-3′ and 1,492R (5′-GGCTACCTTGTTACGACTT-3′). The serotype was determined by the slide agglutination test using *Salmonella* antisera (SSI Diagnostica, Denmark) according to the White–Kauffmann–Le Minor scheme (Issenhuth-Jeanjean et al., 2014).

*Escherichia coli* (*E. coli*) ATCC 25922 was used as the quality control strain for antimicrobial susceptibility testing and *E. coli* J53 (sodium azide resistant) was used as the recipient strain for conjugation. Strains were routinely grown for 12–24 h at 37°C in Luria–Bertani (LB) broth or agar (Guangdong Huankai Microbial Science and Technology Corporation Ltd., Guangzhou, China) supplemented with antibiotics when appropriate.

### Antibiotic susceptibility testing

The isolate was tested for susceptibility to a panel of antimicrobial drugs by disk diffusion method (CLSI, 2017), including amikacin (30 μg), ampicillin (10 μg), amoxicillin/clavulanic acid (20/10 μg), ampicillin-sulbactam sodium (10/10 μg), azithromycin (15 μg), aztreonam (30 μg), cephalosporins IV [cefepime (30 μg)], cephalosporins III [cefotaxime (30 μg) and ceftazidime (30 μg)], cephalosporins II [cefoxitin (30 μg) and cefuroxime (30 μg)], cephalosporins I [cefazolin (30 μg)], chloramphenicol (30 μg), ciprofloxacin (5 μg), doxycycline (30 μg), ertapenem (10 μg), fosfomycin (200 μg), gentamicin (10 μg), imipenem (10 μg), meropenem (10 μg), netilmicin (30 μg), piperacillin (100 μg), streptomycin (10 μg), tigecycline (15 μg), tetracycline (30 μg), tobramycin (10 μg), and trimethoprim/sulfamethoxazole (23.75/1.25 μg) (Hangzhou Microbial Reagent Corporation Ltd., China). Minimal inhibitory concentrations (MICs) of the isolate to ciprofloxacin, nalidixic acid, polymyxin B/colistin, and cefotaxime (Sigma-Aldrich, St. Louis, MO, United States) were determined by broth microdilution method (CLSI, 2017). Production of extended-spectrum β-lactamase (ESBL) was confirmed by the disk diffusion clavulanate inhibition test using ceftazidime and cefotaxime (CLSI, 2017). Results were interpreted according to the Clinical and Laboratory Standards Institute (CLSI) and the European Committee on Antimicrobial Susceptibility Testing (EUCAST) breakpoints (The European Committee on Antimicrobial Susceptibility Testing [ECAST], 2017). The diameter of disks was presented as mean values from replications with SEs. The reference strain *E. coli* ATCC 25922 served as quality control.

An MDR isolate is defined as an isolate demonstrating resistance to three or more antibiotics belonging to different antibiotic classes (Magiorakos et al., 2012). All the measurements were performed in duplicates and each experiment was repeated three times.

### Whole-genome sequencing and annotation

Genomic DNA was extracted using a commercial DNA extraction kit (Magen, Guangzhou, China) following the manufacturer’s recommendations. The whole genome of the isolate was sequenced by combining short-read and long-read technologies. The first set of reads was obtained on the Illumina Hiseq × 10 platform with a 150-bp paired-end reads approach (MajorBio Corporation, Shanghai, China). The second set of reads was obtained on a MinION Nanopore Sequencer (Oxford Nanopore, Oxford, United Kingdom), and a library was prepared with the 1 D ligation approach. The genome of the isolate was assembled *de novo* using both the short- and long-reads with SPAdes version 3.14.0 (Bankevich et al., 2012) and Unicycler hybrid assembler version 0.4.8 (Wick et al., 2017), and annotated by Prokka version 1.14.6 (Seemann, 2014).

Clonal analysis was assessed by multilocus sequence typing (MLST) version 2.0.^1^ The presence of acquired antibiotic resistance genes and mutations in the quinolone resistance-determining regions (QRDR) (*gyrA*, *gyrB*, *parC*, and *parE*) were assessed by ResFinder 4.1 (Zankari et al., 2012), and, subsequently, the genes were extracted from the genome sequences and further confirmed by BLAST nucleotide (BLASTn).^2^ PlasmidFinder version 2.1 and pMLST version 0.1.0 were used to identify plasmid replicon type and sequence type (ST) (Carattoli and Hasman, 2020). The complete plasmid sequence was BLASTn against the nr database with default parameters. Highly similar plasmids were selected for comparison. The map was generated by BRIG 0.95-dev.0004 (Alikhan et al., 2011).

### Phylogenetic analysis of the genomic sequences

In order to assess the relatedness of 16Sal016 with other *S*. Saintpaul strains from different sources and countries, we retrieved all the 2,947 genome sequences of *S*. Saintpaul that have been released from EnteroBase databases (accessed on 8 March 2022) and performed core genome multilocus sequence typing (cgMLST) (cgMLST scheme available on EnteroBase).^3^ A minimum spanning tree was created from cgMLST allelic differences in EnteroBase using GrapeTree with the RapidNJ algorithm (Zhou et al., 2020).

### Conjugation

The transferability of the plasmid was assessed by performing the conjugation experiment using sodium azide-resistant *E. coil* strain J53 as a recipient strain by solid mating on a filter (Whatman, Maidstone, United Kingdom) (Hammerum et al., 2016). Briefly, recipient and donor strains were cultured overnight and harvested, washed with saline, mixed together in a ratio of 1:1, and then spotted onto a 0.45-μm pore size filter (Millipore) on LB plates. They were also spotted individually on LB plates as controls. After overnight incubation at 37°C, mating spots were washed, resuspended in saline, and then serially diluted and plated on LB media containing 150 μg/ml sodium azide and 4, 8, or 16 μg/ml of cefotaxime to select transconjugants. Control spots were transferred to the same selective media to make sure that no growth was observed.

The conjugation frequency was calculated as the ratio of transconjugants over the number of recipients. The transfer of the plasmid was confirmed by PCR targeting *bla*_CTX–M–55_ gene with primer CTX-M-55-F (5′- AAGCACGTCAATGGGACGAT -3′) and CTX-M-55-R (5′- CCTTAGGTTGAGGCTGGGTG -3′), as well as the *uidA* household gene of *E. coli* with the primers U-F (5′-TGGAATTTCGCCGATTTTGC-3′) and U-R (5′-ATTGTTTGCCTCCCTGCTGC-3′) (Heijnen and Medema, 2016). Further, the plasmid DNA was extracted from a selected transconjugant by a commercial plasmid extraction kit (Magen, Guangzhou, China) following the manufacturer’s recommendations, and sequenced on the Illumina Hiseq platform (MajorBio Corporation, Shanghai, China).

### Nucleotide sequence accession number

The Illumina sequence data were deposited in the Enterobase database under the barcode numbers SAL_KB2591AA. The assembly genome sequence of *S*. Saintpaul 16Sal016 was deposited in the National Center for Biotechnology Information (NCBI) database under the Biosample number SAMN12070262.

## Results

### Identification of *Salmonella*

The isolate 16Sal016 was confirmed as *S*. Saintpaul (4,12:e,h:1,2) by biochemical confirmation, 16S rRNA gene sequencing, and serotyping.

### Antibiotic susceptibility

Antibiotic susceptibility testing showed that the isolate 16Sal016 was resistant to ampicillin, azithromycin, aztreonam, cefazolin, cefuroxime, cefotaxime, ceftazidime, chloramphenicol, doxycycline, gentamicin, nalidixic acid, streptomycin, tetracycline, and trimethoprim-sulfamethoxazole, ciprofloxacin (based on the EUCAST clinical breakpoint), intermediate resistant to ampicillin-sulbactam, and produced ESBL. The isolate exhibited MIC values of ciprofloxacin, nalidixic acid, and cefotaxime for 1, 128, and 128 mg/l, respectively.

### General genomic features

The complete genome sequence of *S*. Saintpaul (16Sal016) contained a circular 4,785,442 bp chromosome with G + C content of 52.2%, and a 259,529 bp IncHI2 plasmid, denoted as pSal016. There were 4,736 predicted coding regions (CDs) in the whole-genome sequence. MLST analysis showed that 16Sal016 belonged to ST27.

A total of 15 acquired resistance genes were identified in 16Sal016 by ResFinder, which encodes resistance to nine different antimicrobial classes, including aminoglycoside, beta-lactam, FQs, macrolide-lincosamide-streptogramin B, phenicol, rifamycin, sulfonamide, tetracycline, and trimethoprim (Table 1). All the antimicrobial resistance genes were located on the plasmid.

**TABLE 1:** The antibiotic resistance profile and drug resistance genes of *S*. Saintpaul 16Sal016, the selected transformant (16Sal016T), and the recipient (*E. coli* J53).

	MIC^a^ (mg/L)			Antibiotic resistance genes on plasmid
	
Isolate	CTX	CIP	NAL	
16Sal016	128	1	128	*bla*_*LAP–*2_, *bla*_*TEM–*1*B*_, *bla*_CTX–M–55_, *qnrS1*, *mph(A)*, *lnu(F)*, *tet(A)*, *aph(6)-Id*, *aadA22*, *aac(3)-IId*, *aph(3*′*)-Ia*, *sul3*, *floR*, *arr-2*, *dfrA14*
16Sal016T	128	1	128	*bla*_*LAP–*2_, *bla*_*TEM–*1*B*_, *bla*_CTX–M–55_, *qnrS1*, *mph(A)*, *lnu(F)*, *tet(A)*, *aph(6)-Id*, *aadA22*, *aac(3)-IId*, *aph(3*′*)-Ia*, *sul3*, *floR*, *arr-2*, *dfrA14*
*E. coli* J53	<0.5	0.015	0.25	

^a^CTX, cefotaxime; CIP, ciprofloxacin; NAL, nalidixic acid.

### Comparative analysis of plasmid pSal006

**FIGURE 1:**
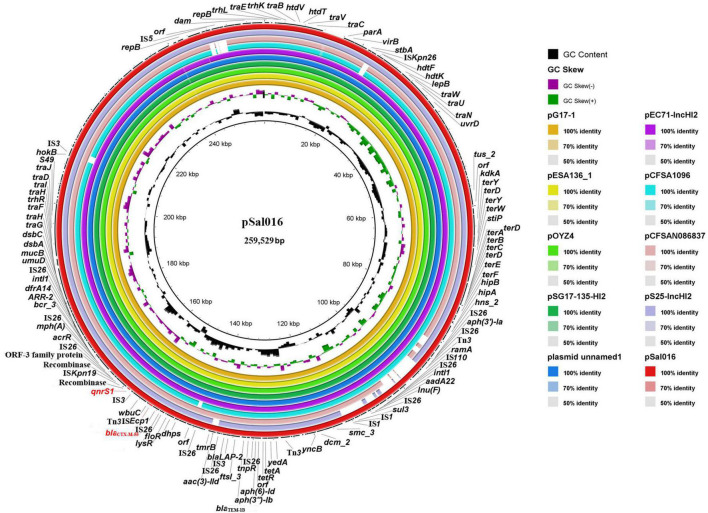
Sequence comparison of plasmid pSal016 identified in *S*. Saintpaul 16Sal016 with similar plasmids in BRIG. GC content and GC skew of pSal016 are depicted in the inner rings. The genes located on pSal016 are annotated at the outside black ring. The *bla*_*CTX–M–55*_ and *qnrS1* genes are marked red.

The pSal016 is a 259,529 bp plasmid, with 112 predicted coding regions (CDs) and an average GC content of 46.7%. Replicon typing of the plasmid by PlasmidFinder and pMLST showed that pSal016 was an IncHI2 plasmid and belongs to ST2. The plasmid includes the core region (*traEBVC*, *traWUN*, and *traGHFIDJ*), which served as the plasmid backbone and is involved in plasmid replication, horizontal transfer, stability, and maintenance functions (Figure 1; Call et al., 2010). Various antimicrobial resistance genes were identified on the plasmid, including *aph(6)-Id*, *aadA22*, *aac(3)-IId*, and *aph(3*′*)-Ia* for aminoglycoside, *mph(A)* for azithromycin, *bla*_*LAP–*2_, *bla*_*TEM–*1*B*_, and *bla*_CTX–M–55_ encoding beta-lactam resistance, *qnrS1* for FQs resistance, *lnu(F)* for lincosamide, *floR* for phenicol, *arr-2* for rifampicin, *sul3* for sulfonamide, *tet(A)* for tetracycline, and *dfrA14* for trimethoprim (Table 1). In addition, copies of transposases and recombinase/integrase family protein were observed on this plasmid (Figure 1).

BLAST nucleotide comparison of the entire plasmid sequence to microbial sequences in GenBank identified six highly similar plasmids with 99–100% sequence coverage and more than 99.9% nucleotide identity and several similar plasmids sharing similar backbones (Figure 1 and Table 2). These plasmids are all the IncHI2 types and belong to ST2, except pCFSA1096 and pCFSAN086837, which belong to ST3. Importantly, the highly similar plasmids were from different hosts than *S*. Saintpaul in different places and years, and most of them were from China (Table 2).

**TABLE 2:** Characteristics of plasmids highly similar to pSal016.

Plasmid name	Replication type	pMLST	Length (bp)	Host	Source	Country (region)	Year	Coverage (%)	Identity (%)	Accession No.
pSal016	IncHI2	2	259,529	*S*. Saintpaul	Fish	China (Guangzhou)	2016	–	–	SAMN12070262
pG17-1	IncHI2	2	264,084	*E. hormaechei*	Duck	China (Henan)	2021	100%	99.98%	CP079936.1
pESA136_1	IncHI2	2	266,043	*E. albertii*	Poultry	China (Shanxi)	2018	100%	99.99%	CP070297.2
pOYZ4	IncHI2	2	257,945	*Salmonella* sp.	Duck	China (Guangzhou)	–	100%	99.96%	MN539018.1
pSG17-135-HI2	IncHI2	2	263,947	*S*. Agona	Australian silver gull chick	Australia	2017	100%	99.98%	CP048776.1
unnamed1	IncHI2	2	263,947	*Salmonella* sp.	Chicken	China (Jiangxi)	2021	100%	99.98%	CP084217.1
pEC71-IncHI2	IncHI2	2	269,592	*E. coli*	Human urine	China (Guangzhou)	2020	99%	99.98%	CP085623.1
pCFSA1096	IncHI2	3	297,348	*S. enterica*	Food	China (Hubei)	2015	97%	100%	CP033347.2
pCFSAN086837	IncHI2	3	255,725	*S*. Newport	Chicken	Viet Nam	2017	94%	99.96%	CP039438.1
pS25-IncHI2	IncHI2	2	237,710	*S*. Saintpaul	Human stool	China (Guangzhou)	2014	93%	100%	CP085697.1
pPJM1	IncHI2	2	263,947	*Salmonella* sp.	Chicken	China (Guangzhou)	2017	90%	99.98%	MN539017.1

### Genetic context of extended-spectrum cephalosporins and fluoroquinolones resistance genes

**FIGURE 2:**
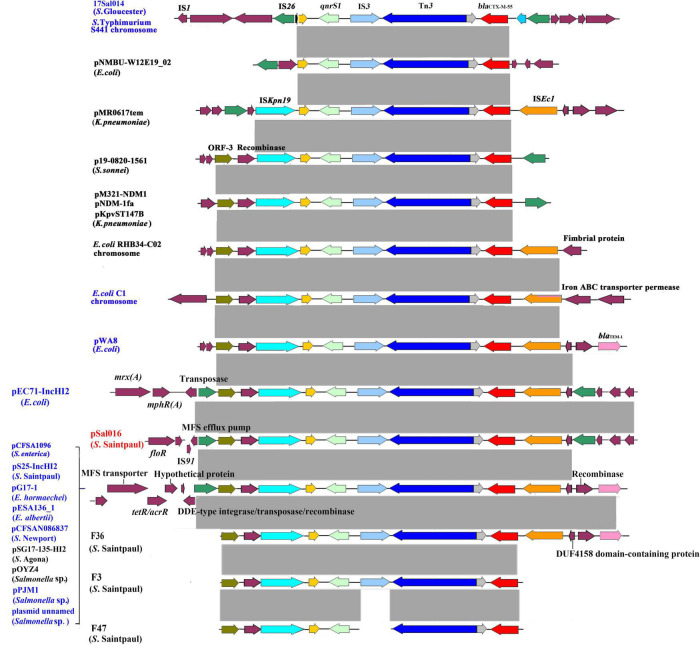
Genetic environment of *bla*_*CTX–M–55*_ and *qnrS1* genes from *S*. Saintpaul isolate 16Sal016 and different bacterial species. The arrows indicate open reading frames. Light gray shading denotes homology regions. Strains from China are in blue font.

The *bla*_CTX–M–55_ and *qnrS1* genes were located on a 12,865 bp IS*26-*mediated composite transposon unit IS*26*-*orf*-*orf*-IS*Kpn19-qnrS1*-IS*3*-Tn*3*-*orf*-*bla*_CTX–M–55_-IS*Ec9*-*orf*-IS*26*. The identical genetic structure was also found on plasmid pEC71-IncHI2 (GenBank accession number CP085623.1) from *E. coli* isolated from a human urine sample in China. Similar structures lacking one of the flanked IS*26* were identified in many other plasmids from different bacterial species, including *E. coli*, *Escherichia albertii*, *Enterobacter hormaechei*, *Salmonella* spp., *S*. Agona, *S. enterica*, *S*. Newport, and *S*. Saintpaul. The core structure *qnrS1*-*orf*-IS*3*-Tn*3*-*bla*_CTX–M–55_ was also observed in many different bacterial species, which can be mediated by IS*26* or IS*Kpn19* (Figure 2). In addition, for comparison, we assembled three *S*. Saintpaul isolates (F36, F3, and F47) reported containing *bla*_CTX–M–55_ and *qnrS1* genes (Nadimpalli et al., 2019; Figure 2).

### Horizontal transfer of the plasmid

Polymerase chain reaction and sequencing results confirmed the successful transfer of the plasmid pSal016 from *S*. Saintpaul 16Sal016 to a plasmid-free recipient *E. coli* J53. Antimicrobial susceptibility testing revealed the acquisition of the plasmid by *E. coli* caused at least 256-fold increase for cefotaxime, 512-fold increase for nalidixic acid, and about 66-fold increase for ciprofloxacin (Table 1). The conjugation rate was 5.4 × 10^–6^ ± 0.6 transconjugant per recipient cell.

### Phylogenetic analysis

**FIGURE 3:**
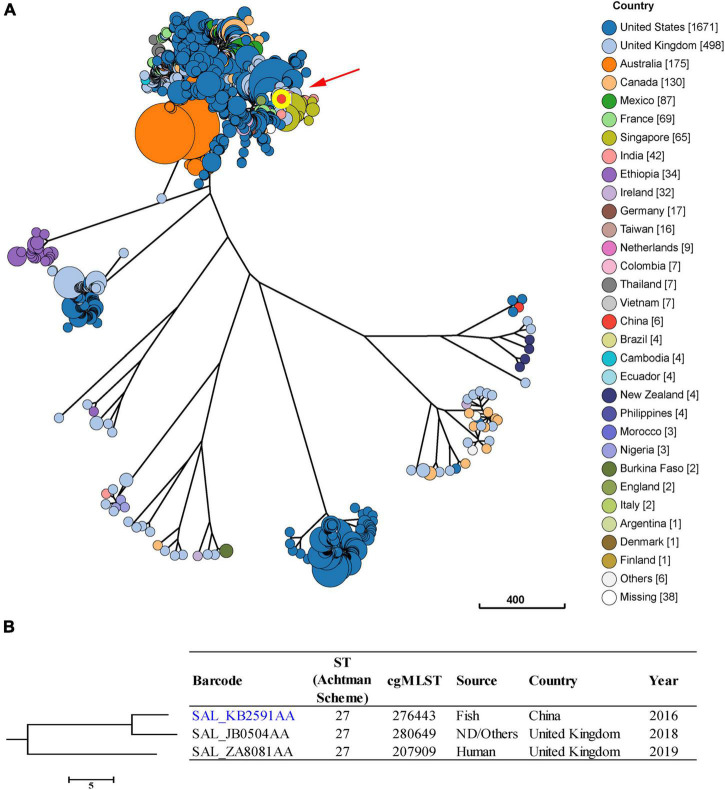
Phylogenetic analysis of 2,947 *S*. Saintpaul isolates from different sources and countries. **(A)** A minimum-spanning tree based on cgMLST analysis. The position of *S*. Saintpaul isolate 16Sal016 is indicated by red arrow and highlighted circle. Each circle represents a cgMLST group and the size of the circle is proportional to the number of isolates in that group. **(B)** Detailed information of strains in the branch contained 16Sal016. The isolate 16Sal016 is marked blue.

Isolates of *S*. Saintpaul were reported to be putatively polyphyletic and characterized by multilineages (Yin et al., 2020). In this study, we found seven major clusters among the 2,947 *S*. Saintpaul isolates from different countries (Figure 3A). These isolates belong to 47 classical MLST types, with the most frequent being ST50 (43.4%), ST27 (29.6%), and ST95 (11.3%) (Supplementary Figure 1).

Core genome multilocus sequence typing and phylogenetic analysis showed that the isolate 16Sal016 harbored a unique cgST profile (Figure 3B and Supplementary Table 1), and displayed the closest relationship to an isolate (Barcode: SAL_JB0504AA) from the United Kingdom in 2018 (Figure 3B). The cgMLST results differentiated the isolate 16Sal016 from the closest related isolate up to the HC5 level (a maximum of 5 cgMLST allelic variations). Isolates belonging to the same HC5 cluster (up to 5 allelic variations between strains) were considered highly probably epidemiologically linked (Bonifait et al., 2021). Therefore, it is inferred that isolate 16Sal016 was highly epidemiologically linked with the isolate from the United Kingdom.

## Discussion

Extended-spectrum β-lactamase-producing and FQs-resistant strains of *Salmonella* have been reported throughout the world, constituting a great public health concern (Castellanos et al., 2018; Nadimpalli et al., 2019; Zhang et al., 2019; Chattaway et al., 2021; Herdman et al., 2021). Contaminated animal-derived food products are an important route of transmission of *Salmonella* from animals to humans (Zhu et al., 2017; Zhang et al., 2018; Wang et al., 2020). In this study, we characterized a Chinese foodborne MDR *S*. Saintpaul isolate, which was found to be resistant to a number of antibiotics, and we further analyzed the genetic context of the corresponding resistance genes, their transferability, and tracked its source by global phylogenetic analysis.

Multidrug-resistant *S*. Saintpaul has been widely reported in many countries (Haq et al., 2017; Ding et al., 2018; Nadimpalli et al., 2019). However, concurrent resistance to ESCs and FQs has only been reported in a *S*. Saintpaul isolate from foals in Pakistan (Haq et al., 2017) and three *S*. Saintpaul isolates from fish in Cambodia (Nadimpalli et al., 2019). In this study, the isolated *S*. Saintpaul strain was found to be co-resistant to ESCs and FQs, as well as multiple antimicrobials, including the most prevalent ACSSuT MDR profile (defined as resistance to ampicillin, chloramphenicol, streptomycin, sulfisoxazole, and tetracycline in *S. typhimurium*) (Mølbak et al., 1999). To the best of our knowledge, this is the first time a foodborne *S*. Saintpaul isolates in China co-resistant to ESCs and FQs has been reported.

The ESCs and FQs resistance in 16Sal016, encoded by *bla*_CTX–M–55_ and *qnrS1* genes, respectively, were located on an IncHI2 plasmid. Several IncHI2 plasmids carried *bla*_CTX–M–55_ gene were found to be closely related to pSal016 and most of them belong to pMLST type 2, suggesting that they are highly similar. It is worth noticing that these plasmids were from different bacterial species and most of them were from China, indicating that the circulating of IncHI2-type plasmid contributes to the increasing prevalence of *bla*_CTX–M–55_ and *qnrS1* genes in China. Importantly, a similar plasmid pS25-IncHI2 was observed to reside in a clinical *S*. Saintpaul isolate collected in the same region, but different year in China. However, phylogenetic analysis of global *S*. Saintpaul isolates based on cgMLST did not reveal any epidemiological links between them, indicating a likely transmission of this plasmid among different *S*. Saintpaul isolates. As evaluated in the present study, pSal016 was transferable to *E. coli*; the conjugation ability of pSal016 may likely contribute to the acquisition of MDR among other bacterial species, which need continuous investigations.

*Salmonella* resistance to ESCs is reported to be associated with cross-resistance to FQs (Lu et al., 2019; Zhang et al., 2019). In our previous study, we identified an IS*26*-mediated composite transposon IS*26*-*qnrS1*-IS*3*-Tn*3*-*orf*-*bla*_CTX–M–55_-IS*Ecp1*-IS*26* that might contribute to the development of co-resistance to ESCs and FQs (Li et al., 2021). In the present study, we found a novel IS*26*-mediated composite transposon IS*26*-*orf*-*orf*-IS*Kpn19-qnrS1-*IS*3*-Tn*3*-*orf*-*bla*_CTX–M–55_-IS*Ec9*-*orf*-IS*26*, as a carrier for *bla*_CTX–M–55_ and *qnrS1* genes in *S*. Saintpaul. An identical structure was also found in *E. coli*, suggesting that it is transferable. Similar structures were widely distributed among different bacterial species. Among them, a uniform unit (*qnrS1*-IS*3*-Tn*3*- *orf*-*bla*_CTX–M–55_) was observed as a core structure, which can be mediated by IS*26* and IS*Kpn19* to transfer.

The global phylogenetic analysis showed that the strain 16Sal016 isolated in 2016 was highly epidemiologically linked with an isolate from the United Kingdom in 2018, suggesting that they might come from the same source. The identification of IS*26*-mediated composite transposon and transferable IncHI2 plasmid carrying *bla*_CTX–M–55_ and *qnrS1* genes in Chinese *S*. Saintpaul represent potential clinical and food safety issues and need to be monitored, since they may transmit to humans through the food chain and may lead to reduced susceptibility of *Salmonella* to frontline drugs of choice for treating severe *Salmonella* infections, such as ESCs and FQs.

## Conclusion

To summarize, this study reports for the first time a foodborne strain of *S*. Saintpaul in China co-resistant to ESCs and FQs and carried *bla*_CTX–M–55_ and *qnrS1* genes. The *bla*_CTX–M–55_ and *qnrS1* genes were located in a novel IS*26*-mediated composite transposon IS*26*-*orf*-*orf*-IS*Kpn19-qnrS1*-IS*3*-Tn*3*-*orf*-*bla*_CTX–M–55_-IS*Ec9*-*orf*-IS*26* on a transferable IncHI2 plasmid. The transfer of the plasmid and the IS*26*-mediated composite transposon may contribute to the dissemination of *bla*_CTX–M–55_ and *qnrS1* genes among different bacterial species and accelerate the development of isolates co-resistant to ESCs and FQs, and this warrants continuous investigations.

## Data availability statement

The datasets presented in this study can be found in online repositories. The names of the repository/repositories and accession number(s) can be found in the article/Supplementary material.

## Author contributions

LL performed the experiment and wrote the manuscript. RO revised the manuscript. JX contributed to reagents, materials, and analysis tools. HM conceptualized and designed the study. SP and LS provided the funding. All authors contributed to the article and approved the submitted version.

## Footnotes

1
https://enterobase.warwick.ac.uk/species/senterica/allele_st_search

2
http://blast.ncbi.nlm.nih.gov/Blast.cgi

3
https://enterobase.warwick.ac.uk
