# Supplementary material for: Genetic context of blaCTX–M–55 and qnrS1 genes in a foodborne Salmonella enterica serotype Saintpaul isolate from China
Source: Front Microbiol. 2022 Aug 9;13:899062. doi: 10.3389/fmicb.2022.899062 (PMC9396133; doi:10.3389/fmicb.2022.899062)

**Supplementary data**

**Supplementary Table S1.** Sequence type (ST) and core genome MLST (cgMLST) profiles of the *S.* Saintpaul isolates that were most closely related to *S.* Saintpaul isolate 16Sal016 in phylogenetic analysis.

| **Barcode** | **ST (Achtman Scheme)** | **Core Genome Sequence Type (cgST)** | **HC0 (indistinguishable)** | **HC2** | **HC5** | **HC**  **10** | **HC**  **20** | **HC**  **50** | **HC**  **100** | **HC200** | **HC**  **400** | **HC**  **900 (ceBG)** | **HC**  **2000 (Super-lineage)** | **HC**  **2600** | **HC**  **2850 (subsp.)** |
| --- | --- | --- | --- | --- | --- | --- | --- | --- | --- | --- | --- | --- | --- | --- | --- |
| SAL_KB2591AA | 27 | 276443 | 276443 | 276443 | 276443 | 268930 | 268930 | 1362 | 1362 | 79 | 79 | 79 | 2 | 2 | 2 |
| SAL_JB0504AA | 27 | 280649 | 280649 | 268930 | 268930 | 268930 | 268930 | 1362 | 1362 | 79 | 79 | 79 | 2 | 2 | 2 |
| SAL_ZA8081AA | 27 | 207909 | 207909 | 207909 | 207909 | 207909 | 207909 | 1362 | 1362 | 79 | 79 | 79 | 2 | 2 | 2 |

HC: Hierarchical clustering

**Supplementary Figure S1.** Distribution of MLST types among 2948 *S.* Saintpaul strains.


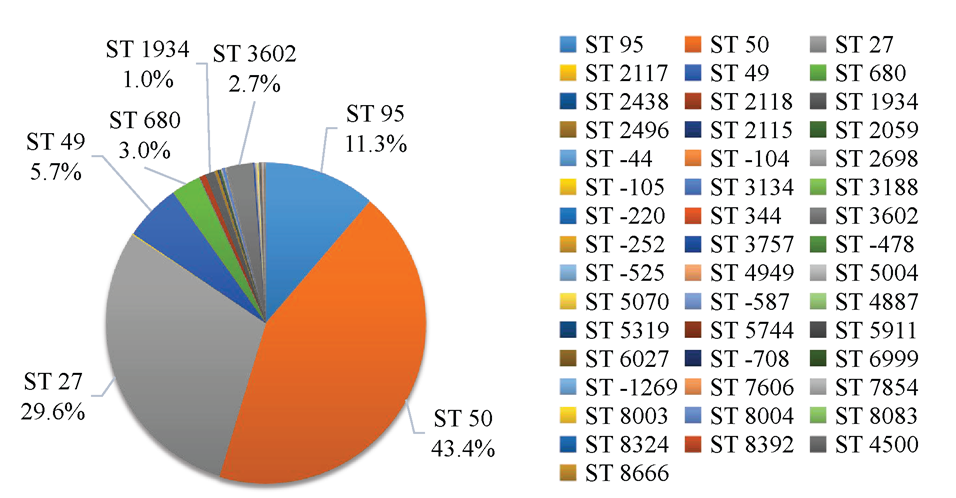

Supplement: Supplementary file 1 [file Data_Sheet_1.docx]
